# Gonadal transcriptome data of fast and slow growth of *Oreochromis niloticus*

**DOI:** 10.1016/j.dib.2026.112525

**Published:** 2026-01-28

**Authors:** Irmawati Irmawati, Dian Novita Sari, Andi Haerul, Alimuddin Alimuddin, Ade Yamindago, Rinto

**Affiliations:** a Faculty of Marine Science and Fisheries, Universitas Hasanuddin, Makassar 90245, Indonesia; b Research and Development Centre for Biotechnology, Institute of Research and Community Service, Universitas Hasanuddin, Makassar 90245, Indonesia; c Marine and Coastal Aquaculture, Faculty of Vocational Studies, Universitas Hasanuddin, Makassar, South Sulawesi 90245, Indonesia; d Department of Aquaculture, Faculty of Fisheries and Marine Sciences, IPB University, Bogor 16680, Indonesia; e Marine Science Study Program, Faculty of Fisheries and Marine Science, Universitas Brawijaya, Malang 65145, Indonesia; f Department of Fisheries Product Technology, Faculty of Agriculture, Universitas Sriwijaya, Indralaya, South Sumatra 30862, Indonesia

**Keywords:** Gonad, Growth, Nile tilapia, Next generation sequencing, Raw data, Transcriptome

## Abstract

*Oreochromis niloticus*, commonly known as Nile tilapia, is an economically significant freshwater fish widely cultured for consumption. Tilapia is one of the most important aquaculture commodities worldwide due to its high demand; therefore, it has been widely cultivated. Here we focus on two strains of Nile tilapia: the **K-strain**, characterized by its rapid growth, and the **N-strain**, known for its slower growth. To enrich the information on the genes involved in growth regulation, RNA sequencing was performed, and transcriptomic datasets were generated from the gonads of both strains. The raw and transcriptomic data have been deposited in the NCBI database, with BioProject Number: PRJNA1354995. These data will provide an essential source of information to enrich the genome annotation of this species, especially in understanding the mechanism underlying the regulation of genes in fast- and slow-growth tilapia.

Specifications Table

**Table:** 

Subject	Biology
Specific subject area	*RNA-seq data of gonads of fast and slow growth of Oreochromis niloticus.*
Type of data	Table Raw, Analyzed, Filtered Data.
Data collection	Samples of male and female *Oreochromis niloticus* from the K22 and N24 strains were collected and preserved in DNA/RNA Shield at −80°C. Total RNA was extracted from three pooled gonadal tissues. RNA sequencing for the four libraries was carried out by PT Genetika Science Indonesia using Illumina NextSeq 2000 platform. The raw reads were filtered using fastp (v0.23.2) and then subsampled into the smallest reads number of reads among samples using seqtk (1.3-r106). The reads quality was checked using fastQC (v0.11.9) and summarized with MultQC (v1.13). The each sample transcripts were quantified sing Hisat2 (v2.2.1) against the reference genome with all gene features (.gtf) information. Mapped reads were counted using htseq (v2.0.2) and the variant calling was conducted using the mapped reads with the GATK pipeline. Variant annotation was performed using snpEff (v5.1d). Enrichment analysis of both Gene Ontology (GO) and Kyoto Encyclopedia of Genes and Genomes (KEGG) was performed using ClusterProfiler (v4.6.2), respectively. Various plots were generated using ggplot2 (v3.4.4).
Data source location	“Polobete Fishfarm” mini hatchery, Pinrang, South Sulawesi, Indonesia (3°53′25.05 S 119°37′56.95 E)*.*
Data accessibility	Repository name: NCBI Data identification number: Bioproject: PRJNA1354995 Direct URL to data: https://www.ncbi.nlm.nih.gov/bioproject/PRJNA1354995/
Related research article	*none.*

## 1 Value of the Data

• Current transcriptome datasets improve the transcriptomic database of fast- and slow- growth of *O. niloticus*.
• These data will contribute to the transcriptomic resources of tilapia, enhancing our understanding of the molecular regulation of growth-related genes.
• These data can be used for comparative analysis between different tilapia growth strains.

## 2 Background

Understanding the molecular mechanisms underlying growth performance in Nile tilapia (*Oreochromis niloticus*) is essential for advancing broodstock improvement programs and selective breeding strategies in modern aquaculture systems. As one of the most widely farmed freshwater fish species globally, tilapia plays a critical role in supporting food security. Previous studies focused on phenotypic performance differences (body weight and length) between pure lines and crossbred generations [1]. Meanwhile in the other studies, liver, muscle, and brain tissues have been used to analyze microRNA profiles and differential gene expression between pure lines and hybrid generations in Nile tilapia, targeting enhanced growth performance and the incorporation of the reddish color phenotype [2,3]. The transcriptomic signatures that differentiate fast- and slow-growth phenotypes only remain insufficiently characterized, particularly at the gonadal level. The gonads function not only in reproduction, but also as key endocrine organs involved in regulating growth-related hormonal pathways, making them a biologically relevant tissue for elucidating gene expression differences between phenotypically divergent strains [4,5].

To address this knowledge gap, we generated high-quality gonadal transcriptome datasets from two genetically distinct growth strains: the fast-growth K strain and the slow-growth N strain. Sequencing using the Illumina NextSeq 2000 platform provided a comprehensive profile of mRNA expression, enabling downstream analyses including functional annotation, differential expression profiling, and pathway enrichment (GO and KEGG). These datasets expand the current genomic resources available for *O. niloticus* and offer valuable reference material for future investigations into growth regulation, molecular breeding, comparative transcriptomics, and the genetic architecture of economically important traits in aquaculture species.

## 3 Data Description

Transcriptomes of fast-growth and slow-growth tilapia were generated from RNA extracts prepared from the gonads of each strain. The number of reads generated ranged from 23,5 to 29,2 million, indicating sufficient sequencing depth for reliable differential gene expression analysis and functional annotation. The mean quality scores ranged from 38,20 to 38,37, reflecting very high base-call accuracy. These results indicated that the sequencing data exhibit a very low error rate and are therefore suitable for downstream analysis. The raw data were deposited in the SRA database with an assigned accession number (Table 1)*.*

**Table 1:** Read number, quality score, and SRA accession number of nile tilapia in this dataset.

Sample	Read number	Mean Quality (%)	SRA Number
Nj_g	23,506,485	38.20	SRX30957877
Nb_g	27,376,758	38.35	SRX30957876
Kj_g	25,267,027	38.36	SRX30957875
Kb_g	29,231,008	38.37	SRX30957874

Samples with quality scores above 30 were classified as high quality [6]. Nj_g = Male gonads of Nile tilapia strain N, Nb_g = Female gonads of Nile tilapia strain N, Kj_g = Male gonads of Nile tilapia strain K, Kb_g = Female gonads of Nile tilapia strain K

After quality filtering, RNA-seq analysis generated between 96,072,184-123,829,388 high-quality clean reads across samples (Table 2). Sequencing quality was high, with Q20 scores ranging from 98.9% to 99.2% and Q30 values between 94.3% to 95.2%. These values indicating excellent sequencing quality and high base-calling accuracy. The GC content ranged from 46% to 50% across samples. These values fall within the expected range for teleost fish transcriptomes. Minor variations among strains may be associated with differences in gene expression profiles rather than methodological artifacts, while direct epigenetic implications cannot be inferred from RNA-seq data alone.

**Table 2:** Statistics of Nile tilapia gonads transcriptome assembly

Parameters	Nj_g	Nb_g	Kj-g	Kb-g
Total reads before filtering	134,707,220	181,797,016	142,171,644	197,124,482
Total reads after filtering	96,072,184	114,550,210	123,829,388	120,339,324
Average unique reads	24,018,046	23,271,232	24,699,505	23,782,834
Average duplicated reads	24,018,046	34,003,873	37,215,190	36,386,829
Total bases	11,565,434,000	13,142,988,000	15,563,405,000	14,319,983,000
Q20 bases	11,438,242,000	13,034,177,000	15,423,664,000	14,196,327,000
Q30 bases	10,907,386,000	12,541,038,000	14,791,575,000	13,634,531,000
Q40 bases	10,907,386,000	12,541,038,000	14,791,575,000	13,634,531,000
GC content	48%	47%	50%	46%
Reads with low quality	26358	26292	14504	23540
Reads with too many N	0	4	32	28
Reads too short	38,608,678	67,220,510	18,327,720	76,761,590
Mean length before filtering	151 bp; 151 bp	151 bp; 151 bp	151 bp; 151 bp	151 bp; 151 bp
Mean length after filtering	122bp, 118bp	115bp, 113bp	126bp, 124bp	120 bp, 117 bp

Notes: Nj_g = Male gonads of Nile tilapia strain N, Nb_g = Female gonads of Nile tilapia strain N, Kj_g = Male gonads of Nile tilapia strain K, Kb_g = Female gonads of Nile tilapia strain K

Short reads were filtered, processed, assembled, and analysed as described in the following sections.

Gene ontology (GO) classification of the Nile tilapia gonads transcripts revealed that annotated transcripts were distributed across the three main GO categories: biological process, cellular component, and molecular function in both comparison groups (Table 3). In the male comparison (Nj_g vs Kj_g), where the Kj_g represents faster growth males, GO terms was relatively evenly distributed among the three categories, indicating a broad involvement of genes associated with cellular activities, molecular interactions, and biological regulation related to growth and gonadal function.

**Table 3:** Gene ontology classification of the Nile tilapia gonads transcripts

Parameters	Number of hits	
	Nj_g vs Kj_g	Nb_g vs Kb_g
Biological process	637	5,910
Cellular component	713	6,306
Molecular function	738	6,177

Although males Nile tilapia exhibited larger body size, the higher number of GO annotations observed in females likely reflects the inherently complex transcriptional regulation of ovarian development and reproductive processes rather than somatic growth. Ovarian tissues are known to require extensive molecular regulation associated with oogenesis and hormonal control, which may explain the elevated functional diversity detected in female gonads. In the female comparison (Nb_g vs Kb_g), a markedly higher number of GO hits was observed across all three categories. This elevated annotation count likely indicates increased functional diversity and regulatory complexity of genes involved in ovarian physiology, rather than body size. The consistent enrichment across GO categories further suggests that the observed differences reflect underlying biological variation rather than technical bias.

## 4 Experimental Design, Materials and Methods

### 4.1 Animal materials

The Nile tilapia K-strain (fast-growth) and N-strain (slow-growth) were obtained from family-based selection stocks at HNK Pasuruan and the Wanayasa collection, respectively, and reared at Polobete Fishfarm, Pinrang, South Sulawesi, Indonesia. Male and female gonads of the K-strain were collected from fish weighing 193.8±4.44 g and 169.53±3.94 g, respectively, whereas male and female gonads of the N-strain were obtained from fish weighing 162.30±4.78 gr and 91.57±6.47 g, respectively. All fish used in this study were five months old. The gonad segments were frozen in DNA/RNA shield (Zymo Research) and stored at -80°C until use.

### 4.2 Total RNA extraction and transcriptome sequencing

Gonadal tissues from four individual fish were pooled to generate one composite sample for each group. Total RNA was then extracted using a commercial RNA extraction kit (TRIzol reagent, Thermo Fisher Scientific), following the manufacturer's instructions. mRNA was enriched from the total RNA using a poly-A enrichment module, which selectively isolates polyadenylated mRNA transcripts according to the manufacturer’s protocol (NEBNext Poly(A) mRNA Magnetic Isolation Module, New England Biolabs). The enriched mRNA was fragmented and hybridized with the Collibri Helper Adaptor Mix, followed by direct adapter ligation. First-strand cDNA was synthesized from the adapter-ligated RNA, and subsequent PCR amplification was performed to generate the final library sample. Library quantification and quality evaluation were performed using a Qubit Fluorometer and an Agilent TapeStation, respectively. Sequencing was performed on the Illumina NextSeq 2000 platform (Illumina Inc., San Diego, CA, USA) using a 300-cycle kit (PE150), according to the manufacturer’s instructions (Illumina Inc.; Invitrogen Collibri Stranded RNA Library Prep Kit, Thermo Fisher Scientific). Sequencing adaptors used were 5’-AGATCGGAAGAGCACACGTCTGAACTCCAGTCA-3’ (forward) and 5’-AGATCGGAAGAGCGTCGTGTAGGGAAAGAGTGT-3’ (reverse).

### 4.3 Raw reads processing and assembly

The quality of raw reads was checked, and low-quality reads were removed using bioinformatics software. Filtering was performed using fastp (v0.23.2) [7]. The filtered reads from each sample were then subsampled into the smallest reads number of reads among samples using seqtk (v1.3-r106) [8]. Quality check of sequencing reads was performed using fastQC (v0.11.9) [9] and summarized with MultQC (v1.13) [10][7]. Transcript quantification for each sample was performed using Hisat2 (v2.2.1) [11] against the reference genome (GCA_001858045.3) with all gene features (.gtf) information. Mapped reads were counted using htseq (v2.0.2) [12]. Variant calling was conducted using the mapped reads with the GATK pipeline [13]. Variant annotation was performed using snpEff (v5.1d) [14]. The statistics of the assembly are shown in Table 2. Enrichment analysis of both Gene Ontology (GO) and Kyoto Encyclopedia of Genes and Genomes (KEGG) was performed using ClusterProfiler (v4.6.2) [15], respectively. Various plots were generated using ggplot2 (v3.4.4) [16]. A summary of the GO analysis results is shown in Gene ontology (GO) classification of the Nile tilapia gonads transcripts revealed that annotated transcripts were distributed across the three main GO categories: biological process, cellular component, and molecular function in both comparison groups (Table 3). In the male comparison (Nj_g vs Kj_g), where the Kj_g represents faster growth males, GO terms was relatively evenly distributed among the three categories, indicating a broad involvement of genes associated with cellular activities, molecular interactions, and biological regulation related to growth and gonadal function.

## Limitations

The differentially expressed genes and enriched pathways identified in this dataset have not been validated through independent experimental approaches, such as quantitative PCR (qPCR), gene knockdown, or functional assays. Consequently, the biological interpretations derived from these results should be considered preliminary and interpreted with appropriate caution*.*

## Ethics Statement

Here we confirming that those experiments complied with the National Institutes of Health guide for the care and use of laboratory animals (NIH Publications No. 8023, revised 1978).

## Credit Author Statement

**Irmawati**: Conceptualization, data curation, investigation, methodology, formal analysis, writing-original draft, review & editing. **Dian Novita Sari**: Investigation, methodology, formal analysis, writing – original draft, review & editing. **Andi Haerul**: investigation, methodology, project administration, writing-editing. **Alimuddin Alimuddin**: conceptualization, supervision, writing – review & editing **Ade Yamindago**: supervision, review & editing. **Rinto**: conceptualization, supervision, review & editing.

## Data Availability

Mendeley DataTilapia breeding program (Original data).
